# The Uses of Water in Modern Medicine

**Published:** 1893-07

**Authors:** 


THE USES OF WATER IN MODERN MEDICINE, by Simon Baruch, M. D. Detroit, Mich., Geo. S. Davis.

This is one of a series of paper covered books issued monthly at $2.50 a year. It gives the technique of the bath as applied in different diseases, with illustrations of the *sheet bath* and *wet pack.*

